# Lingual Traction and the Lateral Decubitus Position Facilitate Awake Fiberoptic Oral Intubation in a Patient With Ehlers–Danlos Syndrome (Hypermobile Type) and Craniocervical Fixation: A Case Report

**DOI:** 10.1155/cria/7693647

**Published:** 2026-10-14

**Authors:** Carlos Ramírez-Paesano, Gabriel Valente-Rivero, Eloymar Rivero-Novoa, Yerlin Andrés Colina-Vargas, Albert Juanola-Galcerán, Gloria Molins

**Affiliations:** ^1^ Servei Central Anestesia (Anestalia) Centro Médico Teknon Grupo Quirónsalud. c/Vilana, 12. Barcelona 08017 Spain teknon.es; ^2^ Hospital Clinic Universitat de Barcelona c/Villarroel, 170 Barcelona 08036 Spain ub.edu

**Keywords:** awake fiberoptic intubation, craniocervical fixation, difficult airway, Ehlers–Danlos syndrome, lingual traction

## Abstract

**Background:**

Awake fiberoptic intubation (AFOI) remains the preferred technique for anticipated difficult airway management. However, severe restriction of mouth opening, fixed craniocervical anatomy, and sedation‐induced hypopharyngeal collapse may substantially impair endoscopic visualization and increase procedural difficulty. Simple mechanical maneuvers that improve upper airway patency may facilitate successful intubation in these highly challenging patients.

**Case Presentation:**

We report the airway management of a 24‐year‐old woman with Ehlers–Danlos‐hypermobile type (EDS‐HT) syndrome, previous craniocervical fusion, severe temporomandibular dysfunction, and a history of a prior *cannot intubate, cannot oxygenate* event requiring emergency tracheostomy. Preoperative evaluation demonstrated extreme anatomical airway restriction, including complete absence of cervical extension, severely limited mouth opening, and ultrasonographic evidence of markedly reduced submandibular compliance (hyomental distance 2.8 cm; hyomental distance ratio 1.0). During the first‐stage spinal surgery, awake fiberoptic orotracheal intubation in the supine position, with dexmedetomidine‐light sedation and local anesthesia, was initially hindered by severe hypopharyngeal collapse. Application of lingual traction immediately restored glottic exposure, allowing successful first‐attempt intubation. Seven days later, during the second‐stage procedure, AFOI was performed in the left lateral decubitus position combined with lingual traction. This approach further improved airway patency, reducing the time to glottic visualization from 15 to 5 s and total intubation time from 90 to 30 s, while maintaining first‐pass success.

**Conclusion:**

This case illustrates that combining lingual traction with lateral decubitus positioning can mechanically optimize upper airway patency during AFOI in patients with fixed craniocervical anatomy and EDS‐HT patients. These simple, low‐cost, and readily reproducible maneuvers may represent valuable adjuncts when conventional airway techniques are limited or contraindicated.

**Highlights**
1. Lingual traction provides immediate mechanical enlargement of the hypopharyngeal space and may rapidly restore glottic visualization when conventional fiberoptic advancement is unsuccessful.
2. Combining lingual traction with lateral decubitus positioning further optimizes upper airway patency by reducing gravity‐dependent collapse of the tongue and pharyngeal soft tissues.
3. Simple, low‐cost mechanical maneuvers should be considered before escalating to more invasive airway interventions in carefully selected patients with anticipated difficult airways such as patients with EDS‐HT with craniocervical fixation.

## 1. Introduction

Awake fiberoptic intubation (AFOI) remains the preferred technique for managing the anticipated difficult airway because it preserves spontaneous ventilation while minimizing cervical spine movement and allowing continuous neurological assessment [1, 2]. Nevertheless, successful AFOI depends on adequate visualization of the upper airway, which may be severely compromised in patients with fixed craniocervical anatomy, restricted mouth opening, or sedation‐induced collapse of the tongue and hypopharyngeal soft tissues.

Mechanical maneuvers that enlarge the upper airway without increasing cervical movement may improve fiberoptic visualization in these challenging situations. Lingual traction, achieved by anterior displacement of the tongue, has been shown to increase hypopharyngeal patency and facilitate fiberoptic intubation in selected difficult airway scenarios [3, 4]. Recent anatomical evidence identifying a tendinous connection between the genioglossus muscle and the epiglottic cartilage provides a physiological explanation for this maneuver, whereby anterior traction on the tongue promotes indirect elevation of the epiglottis and improves glottic exposure [5]. Likewise, body position has an important influence on upper airway mechanics. Lateral decubitus positioning may reduce gravity‐dependent posterior displacement of the tongue and pharyngeal soft tissues, thereby improving airway patency compared with the supine position [6].

Patients with Ehlers–Danlos syndrome (EDS) represent a particularly challenging population for airway management. Connective tissue abnormalities may be associated with temporomandibular dysfunction, restricted mouth opening, cervical instability or previous craniocervical fusion, tissue fragility, mucosal bleeding, and an increased risk of neurological injury during airway manipulation. In addition, repeated surgical procedures and previous difficult airway experiences frequently result in marked anxiety and reluctance to undergo awake airway management, further complicating perioperative care.

Although lingual traction and alternative patient positioning have each been described individually as adjuncts to difficult airway management, reports describing their combined use during AFOI in patients with fixed craniocervical anatomy are exceedingly rare [7, 8]. We present the sequential use of lingual traction alone and, subsequently, lingual traction combined with left lateral decubitus positioning during two staged surgical procedures in a patient with Ehlers–Danlos‐hypermobile type syndrome (EDS‐HT) and previous craniocervical fusion. The case illustrates a simple, inexpensive, and reproducible strategy to mechanically optimize upper airway patency when conventional approaches are severely limited.

## 2. Case Presentation

A 24‐year‐old woman (54 kg, 168 cm) with EDS‐HT syndrome was scheduled for a two‐stage elective T2–T12 posterior spinal fixation for costovertebral joint instability.

Her medical history included mitochondrial disease, severe chronic cervical and thoracic pain, fibromyalgia, postural orthostatic tachycardia (POT) syndrome, diabetes insipidus, adrenal insufficiency, severe temporomandibular joint dysfunction with chronic pain, and previous craniocervical fusion. She also reported marked capillary fragility and recurrent epistaxis, consistent with the underlying connective tissue disorder.

The patient had a documented history of an extremely difficult airway, including a previous *cannot intubate, cannot oxygenate* (CICO) event that required an emergency tracheostomy. The CICO event occurred during anesthetic induction for craniocervical fixation surgery. Examination revealed a well‐healed transverse anterior cervical scar measuring approximately 2‐3 cm, which was mildly adherent to the underlying tissues without evidence of active inflammation or infection.

Preoperative airway assessment demonstrated a Mallampati class IV airway, complete absence of cervical extension (Bellhouse–Doré Grade I), a thyromental distance < 6 cm, a thyrosternal distance < 10 cm, an interincisor distance of 1 cm, and a maximum mouth opening of only 1.8 cm.

Preoperative airway planning included both radiological and ultrasonographic evaluation. Ultrasound demonstrated a hyomental distance (HMD) of 2.83 cm and a hyomental distance ratio (HMDR) of 1.0, indicating complete absence of functional cervical extension (Figure 1). Lateral cervical radiography confirmed extensive craniocervical fusion with complete loss of cervical mobility. The lateral X‐ray shows that the oral, pharyngeal, and laryngeal lines form an acute angle, suggesting difficulty in passing an orotracheal tube (Figure 2A). The patient’s short neck and small body habitus further limited the available space for airway manipulation.

**FIGURE 1:**
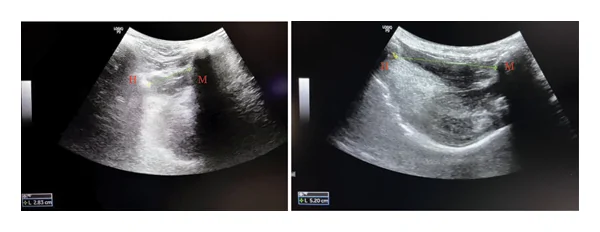
The submandibular ultrasound image on the left shows a hyoid‐to‐mentum distance (HMD) measurement of 2.83 cm. (H) Hyoid bone, (M) mentum. The image on the right shows a patient with a normal HMD (5.20 cm) measured in the neutral position.

**FIGURE 2:**
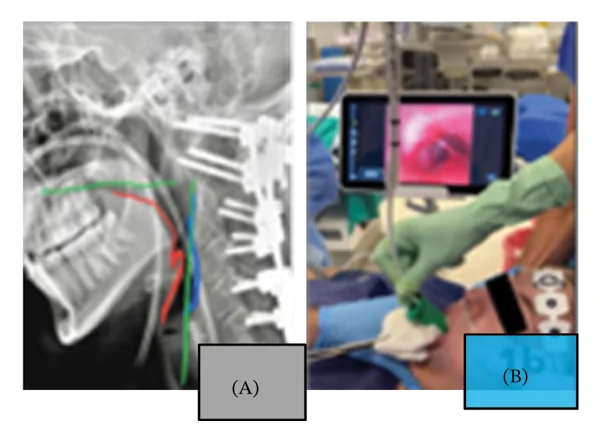
Awake fiberoptic‐guided orotracheal intubation with lingual traction in the supine position. (A) Preoperative lateral cervical radiograph demonstrating craniocervical fusion with superimposed tracings of the oral, pharyngeal, tongue base, and laryngeal axes. (B) Lingual traction‐assisted awake fiberoptic‐guided orotracheal intubation.

Written informed consent was obtained for publication of this case report and the accompanying clinical images. Publication was approved by the Ethics Committee of Centro Médico Teknon.

### 2.1. Timeline (Table 1)

#### 2.1.1. First Procedure (Supine Position)

Standard ASA monitoring and supplemental oxygen (4.0 L/min) delivered via nasal cannula were applied. Airway topicalization consisted of nebulized 5% lidocaine (3 mL), bilateral ultrasound‐guided superior laryngeal nerve blocks (2 mL of 2% lidocaine on each side), and an ultrasound‐guided trans‐cricothyroid injection of 5 mL of 2% lidocaine.

**TABLE 1:** Case report timeline.

Time	Event
Previous years	Hypermobile EDS diagnosed
Previous surgery	Craniocervical fixation
Previous anesthesia	CICO requiring emergency tracheostomy
Preoperative assessment	Ultrasound (HMD 2.83 cm; HMDR 1.0), Lateral X‐ray, difficult airway evaluation
Stage 1 surgery	Awake FOBI + lingual traction (supine)
Day +7 (Stage 2)	Stage 2 surgery: Awake FOBI + lingual traction + left lateral decubitus
Postoperative	Uneventful extubation, ICU, discharge

Sedation was achieved using dexmedetomidine (a loading dose of 1.5 μg/kg administered over 10 min, followed by a continuous infusion of 0.5 μg/kg/h) and fentanyl (1.5 μg/kg). Throughout the procedure, the patient remained calm, cooperative, lightly sedated, and maintained spontaneous ventilation.

A VAMA oropharyngeal airway was inserted, and awake fiberoptic‐guided orotracheal intubation was attempted using a reinforced 6.0‐mm endotracheal tube. Advancement of the bronchoscope was initially hindered by severe hypopharyngeal collapse, preventing identification of the glottic opening. Lingual traction was then applied using a Foster clamp, producing immediate anterior displacement of the epiglottis and restoration of the hypopharyngeal space. This maneuver allowed clear visualization of the glottis and successful first‐pass intubation (Figure 2B).

#### 2.1.2. Second Procedure (Left Lateral Decubitus Position)

Seven days later, the second stage of spinal surgery was performed. Based on the previous experience, AFOI was planned with the patient positioned in the left lateral decubitus position from the outset. Airway topicalization, regional anesthesia, and sedation were identical to those used during the first stage.

After infiltration of the anterior tongue with 1 mL of 2% lidocaine using a 25‐gauge needle, gentle lingual traction was applied using a 2‐0 silk suture. The combination of lingual traction and left lateral decubitus positioning resulted in immediate stabilization of the upper airway and substantially improved fiberoptic visualization. Glottic exposure was achieved in approximately 5 s, compared with 15 s during the first procedure, and total intubation time decreased from 90 to 30 s while maintaining successful first‐pass intubation (Figure 3).

**FIGURE 3:**
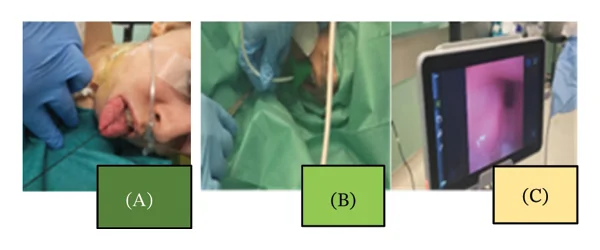
Awake fiberoptic‐guided orotracheal intubation using lingual traction in the left lateral decubitus position. (A) Application of lingual traction. (B) Combined lingual traction and fiberoptic‐guided orotracheal intubation. (C) Fiberoptic view of the vocal cords following optimization of the upper airway.

### 2.2. Postoperative Course

Airway management after both procedures was uneventful. Extubation was performed with the patient in the sitting position. Neuromuscular reversal was not required. A difficult airway cart and equipment for emergency surgical airway access remained immediately available throughout extubation.

No airway‐related complications occurred. The patient maintained stable hemodynamics and oxygenation, and no mucosal bleeding, airway trauma, neurological deterioration, or worsening of temporomandibular joint pain was observed. She was admitted to the intensive care unit for routine postoperative monitoring before being transferred to the surgical ward on postoperative Day 2.

## 3. Discussion

This case demonstrates that combining lingual traction with lateral decubitus positioning can mechanically optimize upper airway patency and facilitate AFOI in a patient with extreme anatomical airway restriction caused by craniocervical fusion and hypermobile EDS. During the first procedure, lingual traction alone immediately restored glottic visualization after severe hypopharyngeal collapse had prevented fiberoptic advancement. During the second stage, adding left lateral decubitus positioning further improved airway patency, resulting in faster glottic identification and a threefold reduction in total intubation time while maintaining first‐pass success.

Based on the previous CICO event, complete absence of cervical extension, severe restriction of mouth opening, and the anticipated difficulty with direct or videolaryngoscopy, awake fiberoptic orotracheal intubation was selected as the primary airway management strategy in accordance with current difficult airway guidelines. The oral route was preferred over the nasal approach because the characteristic mucosal and vascular fragility associated with hypermobile EDS increases the risk of epistaxis, which could obscure the fiberoptic view and jeopardize successful intubation [1, 2, 9].

The principal obstacle to successful AFOI in this patient was not simply restricted mouth opening or cervical immobility but dynamic hypopharyngeal collapse occurring despite preservation of spontaneous ventilation. Even light sedation may reduce upper airway dilator muscle tone, resulting in posterior displacement of the tongue and pharyngeal soft tissues, particularly in patients with severely distorted upper airway anatomy. In this setting, maneuvers that mechanically enlarge the hypopharyngeal space may be more effective than attempting repeated endoscopic manipulation [7, 8, 10, 11].

The effectiveness of lingual traction is supported by recent anatomical evidence demonstrating a tendinous connection between the genioglossus muscle and the epiglottic cartilage. Anterior displacement of the tongue transmits tension to the epiglottis, promoting its elevation and increasing exposure of the laryngeal inlet. Additional contributions from the suprahyoid musculature and hyoid complex probably further enhance upper airway patency [5]. The immediate improvement in glottic visualization observed in both procedures is consistent with these anatomical and biomechanical mechanisms.

Lingual traction has previously been reported to facilitate oral fiberoptic intubation under general anesthesia in a patient with cervical spine fusion, in whom a large epiglottis remained apposed to the posterior pharyngeal wall despite jaw thrust. Anterior traction of the tongue elevated the epiglottis and enabled passage of the endotracheal tube into the trachea [11].

Patient positioning also plays a fundamental role in upper airway mechanics. Compared with the supine position, lateral decubitus reduces gravity‐dependent posterior displacement of the tongue and pharyngeal soft tissues, thereby decreasing airway collapsibility [6]. Although nonsupine positioning has previously been described as an adjunct during awake intubation, reports combining lateral decubitus positioning with lingual traction during AFOI remain exceedingly limited [8]. In our patient, this combination produced noticeably more stable endoscopic conditions and substantially shortened both the time required to identify the glottis and the overall intubation time.

Patients with hypermobile EDS deserve particular consideration because several disease‐specific characteristics may converge to create an exceptionally difficult airway. Restricted mouth opening secondary to temporomandibular dysfunction may preclude insertion of videolaryngoscopes or oral airways, previous craniocervical fusion eliminates compensatory cervical extension, connective tissue fragility increases the risk of mucosal injury and bleeding, and cervical instability raises concern for neurological injury during airway manipulation. In addition, repeated surgical procedures and previous traumatic airway experiences frequently result in significant anxiety and reluctance to undergo awake airway management, making careful communication and patient cooperation essential components of successful airway management [9, 12].

An additional strength of this report is the incorporation of preoperative airway ultrasound. The markedly reduced hyomental distance and an HMDR of 1.0 objectively demonstrated the complete absence of functional cervical extension and the inability to expand the submandibular space during laryngoscopy. Although ultrasound measurements cannot independently predict difficult intubation, they complemented the clinical examination and radiographic findings, reinforcing the anticipated difficulty and supporting selection of AFOI as the safest primary strategy [13, 14]. A history of a “cannot intubate, cannot oxygenate” (CICO) situation requiring an emergency tracheostomy confirms the presence of difficulty with laryngoscopy and tracheal intubation. Several practical implications emerge from this experience. First, lingual traction requires no specialized equipment, is inexpensive, and can be performed rapidly using either a Foster clamp or a traction suture after adequate local anesthesia (Table 2). Second, lateral decubitus positioning may further improve airway patency without increasing cervical manipulation, particularly in patients with fixed craniocervical anatomy. Finally, the sequential use of simple mechanical maneuvers may reduce the need for repeated bronchoscope manipulation, potentially minimizing airway trauma and shortening intubation time.

**TABLE 2:** Pearls and pitfalls of lingual traction during awake fiberoptic intubation.

Pearls	Pitfalls
Easily combined with positioning strategies such as lateral decubitus.	It should not replace the comprehensive airway planning and support strategies recommended in the guidelines.
Can improve the success of the first step when hypopharyngeal collapse does not allow viewing of the glottis in FOB‐guided intubation with analgesic/sedation.	Poor fixation of the tongue (suture or clamp) may lead to loss of traction during the procedure.
Lingual traction is easy to perform and requires minimal equipment.	Excessive traction may cause tongue discomfort or minor mucosal injury if topical anesthesia is inadequate.
Anterior displacement of the tongue increases hypopharyngeal space and improves glottic visualization.	Insufficient airway topicalization may reduce patient tolerance during awake intubation.
Particularly useful in patients with restricted mouth opening, cervical immobility under FOB‐guided intubation.	Lingual traction alone may be insufficient if severe airway collapse persists.

This report has several limitations. As a single case, the findings cannot be generalized to all patients with difficult airways or EDS. Moreover, the observed improvement cannot be attributed exclusively to patient positioning because operator familiarity gained during the first procedure may also have contributed to the shorter intubation time. Nevertheless, the immediate improvement in glottic exposure following lingual traction during both procedures, together with the additional benefit observed in the lateral position, strongly supports a genuine mechanical effect. Prospective studies are warranted to evaluate the reproducibility of this combined approach in patients with fixed cervical anatomy and other forms of anticipated difficult airway.

## 4. Conclusion

This case demonstrates that sequential mechanical optimization of the upper airway using lingual traction and lateral decubitus positioning can substantially improve glottic exposure during AFOI in patients with extreme anatomical airway restriction caused by fixed craniocervical fusion. By relieving hypopharyngeal collapse without increasing cervical manipulation, these simple, inexpensive, and readily reproducible maneuvers facilitated rapid first‐pass intubation in a patient with a previously documented CICO event. Although larger studies are needed to confirm their generalizability, these techniques may represent valuable adjuncts to awake fiberoptic airway management when conventional approaches are technically difficult, ineffective, or potentially hazardous.

## Funding

This research did not receive specific funding.

## Disclosure

All authors have approved the submission and the manuscript has not been published previously and is not under consideration elsewhere.

## Consent

Written informed consent was obtained from the patient for publication of this case report and the accompanying images.

## Conflicts of Interest

The authors declare no conflicts of interest.

## Patient Perspective

The patient reported that the awake intubation was considerably less distressing than her previous airway management experience and expressed satisfaction with the perioperative communication and outcome.

## Supporting Information

Additional supporting information can be found online in the Supporting Information section.

## Supporting information


**Supporting Information** CARE‐Checklist: This case report was prepared in accordance with the CARE (CAse REport) Guidelines and Checklist [15]. The completed CARE Checklist is provided as Supporting Section.

## Data Availability

The data that support the findings of this study are available from the corresponding author upon reasonable request.
