# Supplementary material for: Lingual Traction and the Lateral Decubitus Position Facilitate Awake Fiberoptic Oral Intubation in a Patient With Ehlers–Danlos Syndrome (Hypermobile Type) and Craniocervical Fixation: A Case Report
Source: Case Rep Anesthesiol. 2026 Oct 14;2026:7693647. doi: 10.1155/cria/7693647 (PMC13669532; doi:10.1155/cria/7693647)
Supplement: Supplementary file 1 [file CRIA-2026-7693647-s001.docx]

***Supplementary Section.* CARE-Checklist**

| **CARE Item** | **Description** | **Reported in this manuscript** |
| --- | --- | --- |
| **1. Title** | Identifies the report as a case report and includes the main intervention and condition. | **Yes** |
| **2. Keywords** | Two to five keywords identifying the main elements of the case. | **Yes** |
| **3. Abstract** | Structured summary including background, case presentation, and conclusions. | **Yes** |
| **4. Introduction** | Explains the uniqueness and clinical relevance of the case with supporting literature. | **Yes** |
| **5. Patient Information** | Demographic characteristics, medical history, previous interventions, and relevant comorbidities. | **Yes** |
| **6. Clinical Findings** | Relevant physical examination and airway assessment findings. | **Yes** |
| **7. Timeline** | Chronological summary of the patient's history, interventions, and outcomes. | **Yes *(Table 1)*** |
| **8. Diagnostic Assessment** | Diagnostic methods, imaging, ultrasound findings, clinical reasoning, and anticipated difficult airway assessment. | **Yes** |
| **9. Therapeutic Intervention** | Description of anesthetic management, airway topicalization, sedation, awake fiberoptic intubation, lingual traction, and lateral decubitus positioning. | **Yes** |
| **10. Follow-up and Outcomes** | Postoperative course, airway outcome, complications, and clinical follow-up. | **Yes** |
| **11. Discussion** | Strengths and limitations of the case, comparison with the literature, rationale for management, and clinical implications. | **Yes** |
| **12. Patient Perspective** | Patient's experience and perception of the intervention. | **Yes** |
| **13. Informed Consent** | Written informed consent for publication obtained from the patient. | **Yes** |
